# Outcome of ^177^Lu-PSMA-617 Radioligand Therapy in Chemo-Refractory Patients with Metastatic Castration-Resistant Early-Onset Prostate Cancer

**DOI:** 10.3390/cancers13164193

**Published:** 2021-08-20

**Authors:** Nicolai Mader, Daniel Groener, Nikolaos Tselis, Séverine Banek, James Nagarajah, Frank Grünwald, Amir Sabet

**Affiliations:** 1 Department of Nuclear Medicine, University Hospital Frankfurt, Theodor-Stern-Kai 7, 60590 Frankfurt am Main, Germany; nicolai.mader@kgu.de (N.M.); daniel.groener@kgu.de (D.G.); frank.gruenwald@kgu.de (F.G.); 2 Department of Radiation Oncology, University Hospital Frankfurt, Theodor-Stern-Kai 7, 60590 Frankfurt am Main, Germany; nikolaos.tselis@kgu.de; 3 Department of Urology, University Hospital Frankfurt, Theodor-Stern-Kai 7, 60590 Frankfurt am Main, Germany; severine.banek@kgu.de; 4 Department of Radiology and Nuclear Medicine, Radboud University Medical Center, Geert Grooteplein Zuid 10, 6525 GA Nijmegen, The Netherlands; james.nagarajah@radboudumc.nl

**Keywords:** PSMA, ^177^Lu-PSMA-617, early-onset prostate cancer, metastatic castration-resistant prostate cancer, younger

## Abstract

**Simple Summary:**

The risk of prostate cancer development, the second most commonly occurring cancer in men overall, increases strongly with age. About 10% of patients, however, are diagnosed with early-onset prostate cancer (age at diagnosis: ≤55 years). This is considered to be a distinct clinical and pathological phenotype with a poor prognosis. Generally, prostate cancer cells express high quantities of prostate-specific membrane antigen (PSMA) on their surface. Radioligand therapy is a type of treatment, which, among other available agents, uses the beta-emitting radionuclide ^177^Lutetium (^177^Lu) and a PSMA-targeting ligand termed PSMA-617 for internal irradiation of metastatic prostate cancer cells. The aim of our retrospective study was to assess the efficacy and safety of radioligand therapy with ^177^Lu-PSMA-617 in early-onset metastasized castration-resistant prostate cancer patients refractory to chemotherapy. Special emphasis was placed on the patients’ response to the treatment and survival. The study provides support for the expected shorter survival compared to heterogenous patient groups.

**Abstract:**

The aim of this retrospective study was to assess the outcome of patients with metastasized castration-resistant early-onset prostate cancer refractory to chemotherapy receiving radioligand therapy with ^177^Lutetium-PSMA-617 (LuPSMA-RLT). Twenty-five patients of ≤55 years of age at prostate cancer diagnosis, treated with a median of four (IQR 2–6) cycles (mean of 7.7 ± 1.4 GBq per cycle) every 6–8 weeks, were analyzed. Survival outcome was calculated based on the Kaplan–Meier method. The median progression-free survival (PFS) was 3.8 months (95% CI 2.3–5.3), and overall survival (OS) was 8.5 months (95% CI 6.2–10.8). An initial PSA reduction (≥ 50%) was observed in 9/25 (36%) of patients without being significantly associated with OS (*p* = 0.601). PSA response (PSA decline ≥50% at 12 weeks) was observed in 12/25 (48%) of patients and significantly associated with longer OS (16.0 months, 95% CI 7.4–24.6 vs. 4.0 months, 95% CI 1.1–6.9, *p* = 0.002). Imaging-based response using ^68^Ga-PSMA-11-PET/CT after two to three cycles was seen in 11/25 (44%). Additionally, responders had a significantly longer median PFS (8.7 months, 95% CI 1.3–16.1 vs. 1.9 months, 95% CI 1.7–2.2, *p* < 0.001) and OS (16.0 months, 95% CI 7.6–24.4 vs. 4.0 months, 95% CI 0.9–7.1; *p* = 0.002). Intra- or post-therapeutic toxicity was graded according to the CTCAE v5.0 criteria. Newly developing grade ≥ 3 anemia, leukopenia, and thrombocytopenia occurred in three (12%), one (4%), and three (12%) patients, respectively. One patient showed renal toxicity (grade ≥ 3) during follow-up. Pain palliation (>2 level VAS decline) was achieved in 9/14 (64%) and performance status improvement (ECOG level decline ≥ 1) in 8/17 (47%) of patients. Compared to previous reports, radioligand therapy with ^177^Lu-PSMA-617 in metastasized castration-resistant early-onset prostate cancer patients refractory to chemotherapy yields similar response rates with a comparable safety profile, but is associated with shorter survival.

## 1. Introduction

Prostate cancer is considered a disease of advanced age [1]. Nevertheless, about 10% of patients are initially diagnosed under the age of 55 years [2]. The increasing incidence of prostate cancer, especially in this age group, which may be attributed in part to the more prevalent use of prostate-specific antigen (PSA) testing [3], has led to defining this distinct phenotype with higher cancer-specific mortality as early-onset prostate cancer (HR 1.22 [95% CI 1.10–1.49] compared to patients aged 60–69 years) [2]. Early-onset prostate cancer is known to harbor an aggressive disease course yielding an elevated risk of disease progression and developing metastatic disease [2,4,5]. A large metanalysis on 318,774 prostate cancer patients showed a comparatively lower rate of organ-confined disease in younger patients (compared to an age > 55 years at diagnosis and older, *p* < 0.001) [6]. A large study of 3,006 prostate cancer patients with stage IV disease revealed that metastatic early-onset prostate cancer may also be less responsive to androgen deprivation therapy (ADT) compared to middle-aged (≥56 and ≤65 years) and elderly (≥66 years) patients. Overall survival (OS) at 5 years was significantly worse in the early-onset patient group than that in the middle-aged or elderly group (36.3%, 95% CI 25.1–49.3; 62.1%, 95% CI 57.4–66.6; and 59.8%, 95% CI 57.1–62.5; respectively) [7]. Cancer-specific survival and progression-free survival (PFS) in the early-onset prostate cancer group were also significantly worse compared to the middle-aged and elderly groups (*p* < 0.001 and *p* = 0.002, respectively) [7].

For patients with metastatic prostate cancer failing castration with ADT, only few palliative systemic treatments exist. Taxane-based chemotherapy (i.e., docetaxel and cabazitaxel), new generation antihormonal therapy (i.e., abiraterone acetate and enzalutamide), and, in the presence of symptomatic bone dominant disease, ^223^Radium dichloride constitute the standard treatment options in all age groups [8,9,10,11,12,13]. Previous studies reported a generally less favorable outcome for patients with early-onset prostate cancer (EOPC) at the metastatic castration-resistant stage but a similar response to chemotherapeutic agents [14,15]. The overexpression of prostate-specific membrane antigen (PSMA) in prostate cancer cells is the rationale for radioligand therapy using small-molecule inhibitors with high affinity to PSMA, such as PSMA-617, commonly labelled with beta-emitting ^177^Lutetium (LuPSMA-RLT). Several studies, including the recently published phase III VISION trial, revealed the safety and efficacy of LuPSMA-RLT in metastatic castration-resistant prostate cancer patients after failing at least one line of taxane-based chemotherapy [16]. To date, however, no dedicated analysis of LuPSMA-RLT in metastasized castration-resistant EOPC patients is available.

This study aimed to investigate the safety and efficacy of LuPSMA-RLT in chemo-refractory patients with metastasized castration-resistant EOPC. The impact of response to treatment on survival outcome was of special interest.

## 2. Materials and Methods

### 2.1. Patients

We retrospectively analyzed 25 patients with metastasized castration-resistant EOPC who were treated with ^177^Lu-PSMA-617 in our institution and were followed-up for a minimum of 6 months. All patients received at least one line of taxane-based chemotherapy prior to LuPSMA-RLT, which was performed if all remaining standard treatment options failed or were not applicable. Baseline characteristics are outlined in Table 1. Further eligibility criteria for LuPSMA-RLT included sufficient PSMA-expression in pretherapeutic ^68^Ga-PSMA-11 PET/CT scan, estimated glomerular filtration rate (eGFR) of >30 mL/min/1.73 m^2^, white blood cells ≥ 2.00 × 10^9^/L, platelets ≥ 75 × 10^9^/L, and hemoglobin (Hb) ≥ 8.0 g/dL. Three patients with isolated grade III anemia (7.6, 7.7, and 7.7 g/dL) at baseline and no other treatment alternatives were treated. Next generation sequencing (NGS) was performed in 6/25 patients. Three patients comprised a *BRCA2* point mutation, and one patient showed a *BRCA1* point mutation. Two patients were carriers of an *FGFR4* polymorphism and another two harbored a *TP53* point mutation.

The initiation of treatment was decided by a multidisciplinary tumor board of experts. All patients signed a written informed consent and agreed to the scientific analysis of their data. The retrospective analysis was approved of by a local committee on ethics.

### 2.2. ^177^Lutetium-PSMA-617 Radioligand Therapy

LuPSMA-RLT was performed every 6–8 weeks with a standard activity of 7.4 GBq and was modified when necessary. The PSMA-617 ligand was provided by ABX (Advanced Biochemical Compounds GmbH, Radeberg, Germany) and labelled in-house with ^177^LuCl_3_ (ITM Isotopen Technologien München AG, Garching/Munich, Germany) as described in detail previously [17,18]. In order to limit xerostomia, salivary glands were cooled with ice packages for 2 h beginning 30 min before the administration. ^177^Lu-PSMA-617 was administered intravenously in 30–60 s preceded and followed by 1000 mL 0.9% NaCl solution infusion. The distribution of the radionuclide was recorded using planar whole-body scintigraphy at 24 h and 48 h p.i. All treatment cycles were carried out as in-patient procedures at the nuclear medicine therapy ward.

### 2.3. Response Assessment

PSMA-based imaging response was performed every 2–3 cycles using the recent consensus on ^68^Ga-PSMA-11 PET/CT imaging response assessment [19]: partial response (PR, reduction of uptake and tumor PET volume by >30%), stable disease (SD, uptake and tumor PET volume ± ≤30%; no new lesions), and progression (PD, appearance of >2 new lesions or uptake or tumor PET volume ≥30% increased). Prostate-specific antigen (PSA) changes were classified as: response (≥50% decline 12 weeks after treatment initiation), progression (≥25% increase exceeding 2 ng/mL, confirmed by a second measurement ≥ 3 weeks apart, according to the PCWG3 criteria [20]), and stable (values between <50% decline and <25% increase). Initial PSA change after one cycle was not considered for response assessment if it was not accompanied by other confirming evidence. The visual analog scale (VAS; ranging from 0 to 10) and Eastern Cooperative Oncology Group (ECOG) scale were assessed at baseline and at each treatment cycle and used to evaluate the pain and performance status.

### 2.4. Toxicity Assessment

Hematological (hemoglobin (Hb), white blood cells (WBC), platelets (PLT)) and renal (eGFR) toxicity evaluation was performed through a blood workup routine at baseline, prior to each therapy cycle, 2–4 weeks after each cycle and in 6–12 week intervals throughout follow-up. The severity of adverse events was graded based on the Common Terminology Criteria for Adverse Events (CTCAE), version 5.0, with grade ≥ 3 toxicities deemed significant. Mouth dryness was evaluated at every LuPSMA-RLT cycle using a modified, patient self-reported eight-item xerostomia questionnaire [21].

### 2.5. Data Analysis

Statistical analyses were performed using SPSS software (IBM SPSS Statistics 27.0, Armonk, NY, USA). The significance level was set two-sided at *p* < 0.05. The presentation of the results was conducted either as a median with interquartile range (IQR) or mean ± standard deviation for continuous variables, and categorical variables were presented as frequencies with respective percentages. The Paired-Samples T Test was used to compare intraindividual changes in continuous biochemical parameters. Imaging-based PFS was defined as the time interval from LuPSMA-RLT initiation to the date of the first progression, or death, if no imaging-based progression occurred (defined according to the PCWG3 criteria) [20]. Overall survival (OS) was defined as the time from treatment initiation to death from any cause; censoring was carried out if the patient was alive at the time of analysis. PFS and OS were calculated based on the Kaplan–Meier method (log-rank testing).

## 3. Results

Twenty-five patients with metastasized castration-resistant EOPC were treated with LuPSMA-RLT for a median of four (IQR 2–6) cycles and 7.7 ± 1.4 GBq per cycle reaching a cumulative activity of 31.3 ± 21.3 GBq.

### 3.1. Response

**Figure 1:**
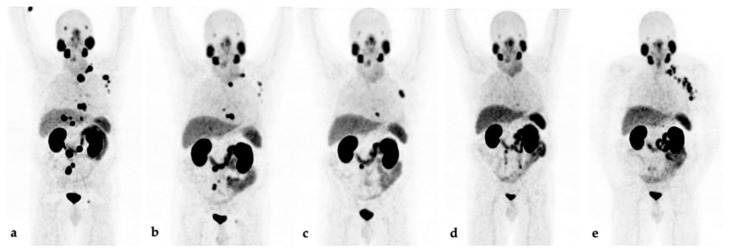
Maximum-intensity projections of ^68^Ga-PSMA-11 PET/CT imaging of a metastasized castration-resistant EOPC patient during LuPSMA-RLT and follow-up. (**a**) At baseline, PSA: 69.3 ng/mL; (**b**) after three cycles, PSA at 12 weeks: 6.8 ng/mL; (**c**) after six cycles, PSA: 2.4 ng/mL; (**d**) after eight cycles, PSA: 0.3 ng/mL; (**e**) restaging 7 months later, PSA: 15.9 ng/mL.

PSA decline (≥50%) after 12 weeks occurred in 12/25 (48%) patients; 6/25 (24%) remained stable. Seven (28%) patients showed PSA progression (*n* = 4) or died (*n* = 3) within 12 weeks. Imaging assessment showed PR in 11/25 (44%) patients, and 4 patients (16%) had SD. Ten (40%) patients had PD (*n* = 7) or were deceased before the assessment (*n* = 3). An example of a disease course on ^68^Ga-PSMA-11 PET/CT imaging is provided in Figure 1.

Among 14 (56%) patients with moderate to severe pain at baseline (defined as ≥4 pain level on a VAS assessment), 9/14 (64%) reported significant improvement of the overall pain (>2 level VAS decline) during LuPSMA-RLT or follow-up. Of 17 (68%) patients with ECOG level ≥ 2 at baseline, an improvement in performance status (ECOG level decline ≥ 1) was observed in 8/17 (47%).

### 3.2. Survival

**Figure 2:**
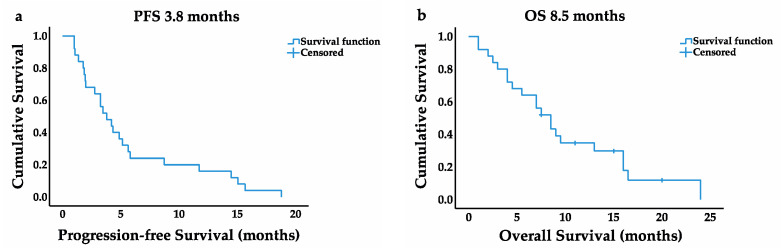
Kaplan–Meier survival curves. (**a**) Progression-free survival in months; (**b**) overall survival in months.

Mean follow-up was 8.7 ± 6.1 months. Twenty-one (84%) patients died by the time of this analysis. Median imaging-based PFS was 3.8 months (95% CI 2.3–5.3), and median OS was 8.5 months (95% CI 6.2–10.8). The Kaplan–Meier survival curves are displayed in Figure 2.

**Figure 3:**
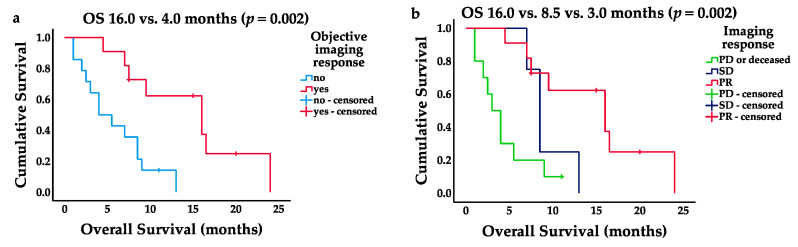
Kaplan–Meier survival curves. (**a**) Objective imaging response (PR) and respective OS in months; (**b**) PR, SD, and PD imaging response and respective OS in months.

Objective imaging response was associated with a significantly longer median OS (PR: 16.0 months, 95% CI 7.6–24.4 vs. SD/ PD or deceased: 4.0 months, 95% CI 0.9–7.1, *p* = 0.002; Figure 3a). Three patients died before the first PSMA-based imaging response assessment. The median overall survival of the remaining 22 patients was: PR (16.0 months, 95% CI 7.6–24.4, *p* = 0.002), SD (8.5 months, 95% CI 7.2–9.8), and PD or deceased (3.0 months, 95% CI 1.5–4.6; Figure 3b). In patients achieving disease control (PR and SD, *n* = 15), the median PFS was 5.6 months (95% CI 4.5–6.8), and the median OS of 13.0 months (95% CI 5.0–21.0) was significantly longer compared to non-responders (3.0 months, 95% CI 1.5–4.6, *p* = 0.001).

**Figure 4:**
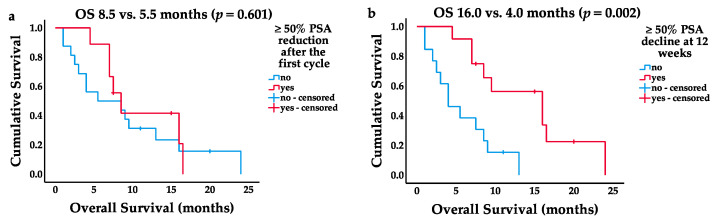
Kaplan–Meier survival curves. (**a**) PSA decline (≥50%) after the first cycle and respective OS in months; (**b**) PSA decline (≥50%) 12 weeks after LuPSMA-RLT initiation and respective OS in months.

An initial ≥ 50% decline in PSA levels was observed in 9/25 (36%) patients with no significant contribution to a longer median OS (8.5 vs. 5.5 months, 95% CI 6.1–10.9, *p* = 0.601; Figure 4a). PSA decline (≥ 50%) 12 weeks after LuPSMA-RLT initiation (*n* = 12) was significantly associated with a longer median OS (16.0 months, 95% CI 7.4–24.6 vs. 4.0 months, 95% CI 1.1–6.9, *p* = 0.002; Figure 4b).

### 3.3. Safety

At baseline, 23 (92%) patients had anemia (16 grade 1, 4 grade 2, 3 grade 3), 1 patient had (4%) leukopenia (1 grade 1), 5 patients had (20%) thrombocytopenia (4 grade 1, 1 grade 2), and 4 patients had (16%) renal function grade 2. Hb, WBCs, and PLTs showed a minor yet significant absolute decline through the course of LuPSMA-RLT. Mean Hb changed from 10.7 ± 1.8 to 10.2 ± 1.9 g/dL (*p* < 0.001), WBCs from 6.4 ± 1.8 × 10^9^/L to 5.0 ± 1.7 × 10^9^/L (*p* < 0.001), and PLTs from 262 ± 92 × 10^9^/L to 182 ± 88 × 10^9^/L (*p* = 0.008).

Newly developing grade ≥ 3 hematological toxicity after at least one cycle of LuPSMA-RLT or during follow-up was observed: significant anemia (grade ≥ 3) in three (12%) patients, leukopenia in one patient (4%), and thrombocytopenia (grade ≥ 3) in three patients (12%). During the follow-up period, grade ≥ 3 anemia, leukopenia, and thrombocytopenia were reversible in 1/5 (20%) patients, 1/1 (100%), and 1/3 (33%), respectively.

Nephrotoxicity (grade ≥ 3) occurred in one (4%) patient, which was reversible during follow-up. We found no significant xerostomia during LuPSMA-RLT and the follow-up period; however, grade 1 or 2 xerostomia began in six (24%) of the patients after a median of two (IQR 1–4) cycles. A summary of the adverse events is shown in Table 2.

## 4. Discussion

This retrospective study is the first report addressing the outcome of patients with metastatic castration-resistant early-onset prostate cancer treated with LuPSMA-RLT. The observed biochemical response rate of 48% (12/25) at 12 weeks is similar to the findings of previous studies on patients with different ages at diagnosis, and responders identified by PSMA-based imaging benefitted from a significantly longer overall survival with a median of 16.0 months (*p* = 0.002) [16,22,23]. Disease control was achieved in 60% (15/25), but the median progression free survival of 5.6 months was relatively short [16,22,23].

In the most recent phase III VISION trial, Sartor et al. showed an imaging PR response rate of 40% using RECIST criteria and bone scan [16]. Using the same criteria, Hofman et al. reported an objective response rate of 49% in the phase II TheraP trial [22]. Concordantly, 44% (11/25) of the patients in our study showed a PR in PSMA-based imaging. Our biochemical response rate (PSA-decline ≥ 50% at 12 weeks) was also comparable to the findings of the VISION trial (48% vs. 46%) [16]. The prognostic relevance of initial PSA-decline of ≥50% at 4 weeks after the first cycle of LuPSMA-RLT is unclear [24,25]. In our study, 9/25 (36%) patients with an initial PSA decline ≥ 50% tended to live longer, but the difference was not statistically significant (8.5 vs. 5.5 months, *p* = 0.601). Similarly, in the WARMTH multicenter study by Ahmadzadehfar et al., PSA response to the first cycle measured after 8 weeks had no significant impact on overall survival (PSA response ≥ 50%: 41.5%, *p* = 0.6) [23].

The response to LuPSMA-RLT seems to be independent of age at diagnosis, yielding similar imaging and biochemical response rates in patients with metastasized castration-resistant EOPC and other age groups. However, the median PFS of 3.8 months (95% CI 2.3–5.3) in our study group is comparatively short, indicating an earlier progression in patients with metastasized castration-resistant EOPC. The VISION trial reported an imaging PFS of 8.7 months for the patients in the ^177^Lu-PSMA-617 plus Standard Care arm, and the TheraP trial showed a PFS of 5.1 months in the experimental group receiving ^177^Lu-PSMA-617 [16,22]. This difference in PFS may have been partly caused by the higher diagnostic ability of ^68^Ga-PSMA-11 PET/CT imaging used for treatment assessment in our study as compared to computer tomography plus bone scan [26]. On the other hand, a study by Barber et al. investigating the influence of previous chemotherapy on the outcome of patients receiving LuPSMA-RLT using ^68^Ga-PSMA-11 PET/CT for response assessment showed a longer median PFS in both groups compared to our study on metastasized castration-resistant EOPC [27]. The median PFS in chemo-refractory patients was 6.0 months (vs. 8.8 months in chemo-naïve) with a mean age of 69 years at treatment initiation. Furthermore, few reports suggest an association between younger age at initiation of LuPSMA-RLT and poor outcome. Heck et al. studied 100 patients with a median age of 72 years (IQR 66–76) and found a significant decrease in the risk of progression (HR 0.7, 95% CI 0.5–0.9, *p* = 0.01) and death of any cause (HR 0.7, 95% CI 0.5–1.0, *p* = 0.07) with every 10-year increase in age [28]. This may be the result of more metastasized castration-resistant EOPC among patients with younger age at the time of treatment initiation.

In our study investigating patients younger than 55 years old at diagnosis, the median age at treatment initiation was 62 (IQR 57–71). Metastasized castration-resistant EOPC patients with disease control by PSMA-based imaging during the course of treatment lived significantly longer than non-responders (13.0 months, 95% CI 5.0–21.0 vs. 3.0 months, 95% CI 1.5–4.6; *p* = 0.001). However, the median OS of 8.5 months in the whole study group was shorter comparing to the reported median OS in the prospective trials with significantly higher ages at treatment initiation, such as in the VISION trial (OS, 15.3 months; age, 70 years), LuPSMA trial (OS, 13.5 months; age, 71 years), and RESIST-PC trial (OS, 14 months; age, 74 years), although the age at diagnosis was not presented [16,29,30]. Similar median ages at the treatment initiation, ranging from 70 to 75 years, were also reported by the majority of the previous retrospective studies on patients treated with LuPSMA-RLT [31,32,33,34,35]. Although the ages at diagnosis were not explicitly displayed, the significantly older cohorts at LuPSMA-RLT initiation imply the inclusion of a relatively small proportion of metastasized castration-resistant EOPC patients with a reportedly distinguished, more aggressive course of disease resulting in shortened sojourn times [2,4,36].

A stronger genetic component with a characteristically higher incidence of germline alterations such as *TMPRSS2–ERG* fusions and *BRCA1/2* mutations has been considered responsible for the poor differentiation and higher mortality [37,38,39,40,41,42]. A study on 333 consecutive castration-resistant prostate cancer patients revealed an OS of 5.5 years (95% CI 3.0–7.5) in CR-EOPC patients compared to 6.7 years (95% CI 5.9–8.4) in those aged 55–64 years, and 7.8 years (95% CI 6.6–9.3) in the age group of 65–74 years at prostate cancer diagnosis, respectively [15]. In six patients with NGS analysis in our study, four patients were carriers of *BRCA1* (*n* = 1) / *BRCA2* (*n* = 3), *TP53* (*n* = 2) point mutations, and *FGFR4* (*n* = 2) polymorphism. *BRCA2* mutations may also lead to more rapid taxane resistance in metastasized castration-resistant prostate cancer patients [43]. A large study on 2019 patients with prostate cancer showed that BRCA1/2 status is an independent prognostic value for OS (HR 1.9; 95% CI 1.1–3.3; *p* = 0.012) [44].

The rate of significant toxicity in our study is comparable to that of previous studies on heterogenous patients. Hematotoxicity was the most frequent adverse event with newly appearing grade 3 anemia during LuPSMA-RLT or during follow-up in three (12%) patients, grade 3 leukopenia in one (4%) patient, and grade 3 thrombocytopenia in three (12%) patients. These findings are in concordance to the VISION trial with grade ≥ 3 anemia occurring in 12.9%, grade ≥ 3 leukopenia in 2.5%, and grade ≥ 3 thrombocytopenia in 7.9% [16]. In the phase II LuPSMA trial by Hofman et al., grade ≥ 3 anemia occurred in 13%, grade ≥ 3 leukopenia in 1%, and grade ≥ 3 thrombocytopenia in 13% [29]. In our study, one patient with a renal impairment at baseline (grade 2) experienced a reduction to grade 3 due to a complicated suprapubic urinary catheter and coagulum formation in the urinary bladder. Renal function returned to the baseline level after appropriate anti-inflammatory treatment. In line with previous studies reporting the renal safety of RLT with LuPSMA-RLT, no significant treatment-induced nephrotoxicity was observed in our patient group [45,46].

The main limitations of our study are the small sample size and its retrospective nature, which inevitably weaken the comparability with the prospective trials and the conclusions drawn. The short survival of the patients limited the follow-up period in our study, preventing the assessment of long-term renal safety. To improve the outcome of RLT, combination therapy by adding antiandrogen therapies or chemotherapeutic agents to LuPSMA-RLT, or by using the tandem approach with ^225^Actinum-PSMA-617/^177^Lutetium-PSMA-617, may be worth considering. Moreover, genetic sequencing was only performed in a minority of patients. Considering the strong genetic component in patients with early-onset prostate cancer, future studies focusing on genetic profiling may provide useful information that can help personalize LuPSMA-RLT.

## 5. Conclusions

The response to radioligand therapy with ^177^Lu-PSMA-617 seems to be independent of the presence of early-onset prostate cancer (EOPC); however, the less favorable survival outcome observed in our study may indicate a more aggressive disease course. Expectedly, the safety of LuPSMA-RLT does not seem to be compromised in metastasized castration-resistant EOPC patients. Comparative studies on age at diagnosis of prostate cancer and the patients’ outcome after LuPSMA-RLT are needed.

## Figures and Tables

**Table 1:** Baseline characteristics.

Variable	All Patients (*n* = 25)
Age at diagnosis, years	54 (51–55)
Age at LuPSMA-RLT initiation, years	62 (57–71)
Time to CR, years	4.5 (1.3–13.8)
Time to LuPSMA-RLT initiation after diagnosis, years	8.3 (4–13.9)
Gleason score *:	
<8	8 (38)
≥8	13 (62)
PSA at LuPSMA-RLT initiation (ng/mL)	179 (69.5–1142)
PSA Doubling Time, months	2 (1.2–3.4)
Hemoglobin (g/dL)	11 (9.5–11.9)
White blood cells (10^9^/L)	6.2 (5.1–7.2)
Platelets (10^9^/L)	276 (217–335)
eGFR (mL/min/1.73 m^2^)	95.3 (72.5–103.8)
Alkaline phosphatase (U/L)	235 (124–568.5)
Lactate dehydrogenase (U/L)	315 (268–509.5)
Disease involvement:	
Bone metastases	24 (96)
Lymph node metastases	20 (80)
Visceral metastases	11 (44)
hepatic	7 (28)
pulmonary	4 (16)
Primary	6 (24)
VAS:	
<4	11 (44)
≥4	14 (56)
ECOG performance status:	
<2	8 (32)
≥2	17 (68)
Previous treatment:	
Abiraterone	24 (96)
Enzalutamide	18 (72)
^223^Radium dichloride	5 (20)
Docetaxel	25 (100)
Cabazitaxel	15 (60)
Nontaxane chemotherapy **	4 (16)

Data presented as median with interquartile range (IQR), or *n* (%), LuPSMA RLT: ^177^Lutetium-PSMA-617 radioligand therapy, CR: castration resistance, PSA: prostate-specific antigen, eGFR: estimated glomerular filtration rate, VAS: Visual Analogue Score, ECOG: Eastern Cooperative Oncology Group, *: for available patients (*n* = 21), ** including one or more regimens: cisplatin, etoposide, 5-FU, carboplatin AUC 5, oxaliplatin, capecitabine, mitoxantrone.

**Table 2:** Hematologic, renal, and salivary gland toxicity grades based on CTCAE v5.0. at baseline and after at least one cycle of LuPSMA-RLT or during follow-up.

Toxicity	Baseline, *n* (%)		Post-Therapeutic, *n* (%)	
	Grade 1/2	Grade 3/4	Grade 1/2	Grade 3/4
Anemia	20 (80)	3 (12)	20 (80)	5 (20)
Leukopenia	1 (4)	0 (0)	12 (48)	1 (4)
Thrombocytopenia	5 (20)	0 (0)	7 (28)	3 (12)
eGFR *	4 (16)	0 (0)	7 (28)	1 (4)
Xerostomia	0 (0)	0 (0)	6 (24)	0 (0)

* Estimated glomerular filtration rate.

## Data Availability

The datasets analyzed and/or analyzed during the current study are available from the corresponding author on reasonable request.
